# Supplementary material for: Hidden microalgae diversity in reef systems: reanalysis of coral microbiomes reveals spatial patterns of coral-associated plastid communities in the Southwestern Atlantic Ocean (SWAO)
Source: PeerJ. 2025 Nov 3;13:e20116. doi: 10.7717/peerj.20116 (PMC12591052; doi:10.7717/peerj.20116)
Supplement: Supplemental Information 1 — The workflow starts with a bibliographic review to compile coral-associated 16S rRNA sequence datasets from the Southwestern Atlantic Ocean (SAO), including sequences derived from bacterial cloning, shotgun, and metabarcoding studies. Preprocessing for the retrieved sequences and other bioinformatic processes are illustrated in hexagons. Classified plastid sequences and their associated metadata are compiled into the SAO plastid inventory (Table S2), enabling further ecological analyses. Two main analysis were conducted: the prevalence assessment of plastid-bearing eukaryotes with in-situ samples from the inventory and a reanaylisis of Mussismilia harttii plastidiomes from Abrolhos. Ecological analyses are illustrated in circles. [file peerj-13-20116-s001.pdf]

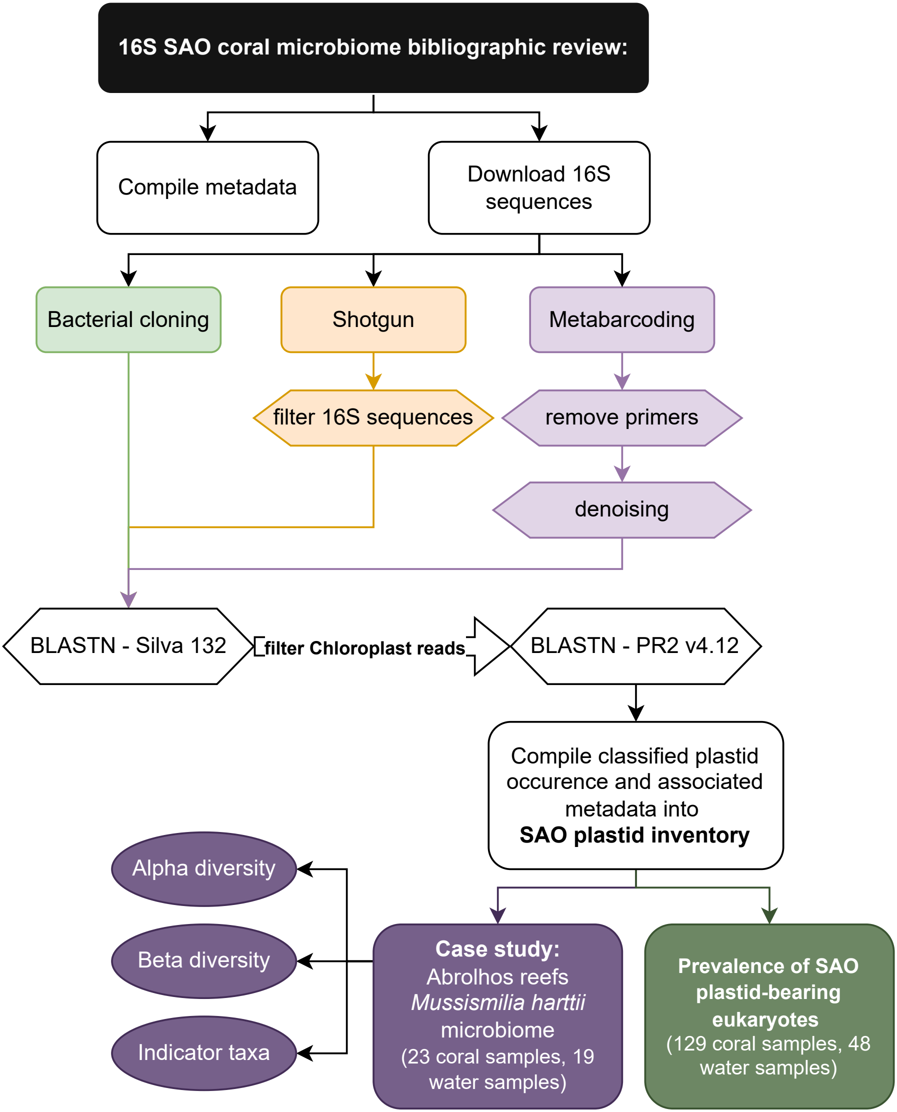

16S SAO coral microbiome bibliographic review:
Compile metadata
Download 16S sequences
Bacterial cloning
Shotgun
Metabarcoding
filter 16S sequences
remove primers
denoising
BLASTN - Silva 132
filter Chloroplast reads
BLASTN - PR2 v4.12
Compile classified plastid occurence and associated metadata into
SAO plastid inventory
Alpha diversity
Beta diversity
Indicator taxa
Case study:
Abrolhos reefs
Mussismilia harttii
microbiome
(23 coral samples, 19 water samples)
Prevalence of SAO plastid-bearing eukaryotes
(129 coral samples, 48 water samples)
