# Supplementary figures and images for: Hidden microalgae diversity in reef systems: reanalysis of coral microbiomes reveals spatial patterns of coral-associated plastid communities in the Southwestern Atlantic Ocean (SWAO)
Source: PeerJ. 2025 Nov 3;13:e20116. doi: 10.7717/peerj.20116 (PMC12591052; doi:10.7717/peerj.20116)

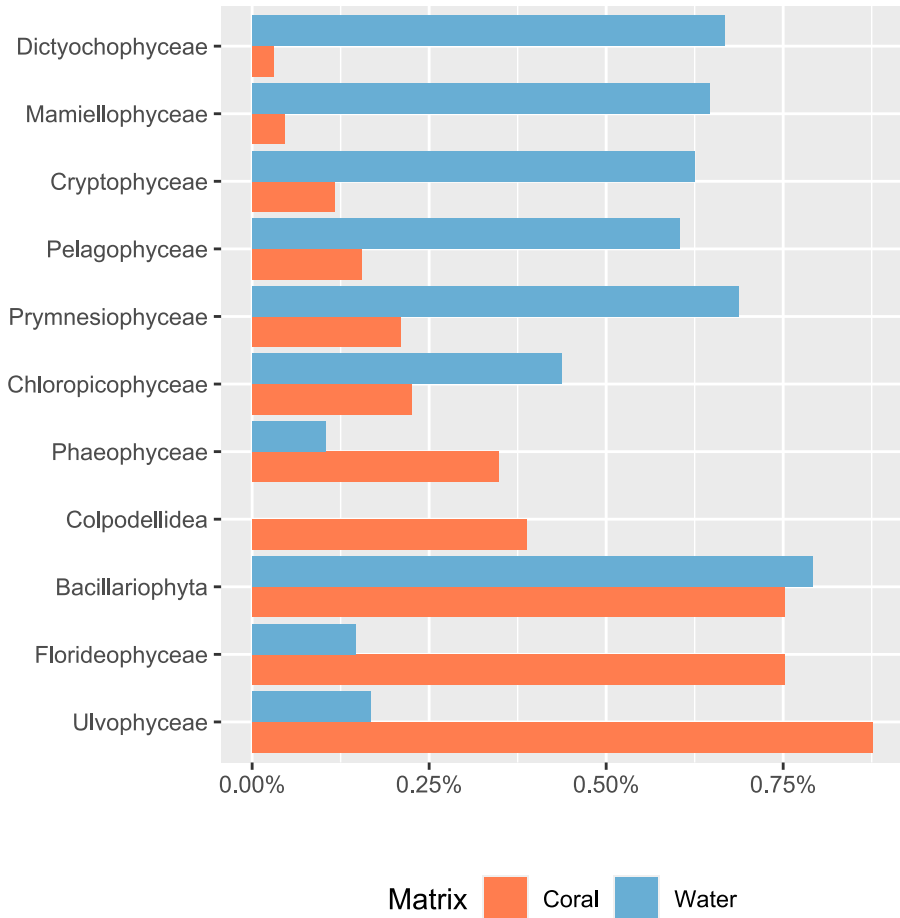

Supplement: Supplemental Information 2 [file peerj-13-20116-s002.pdf]

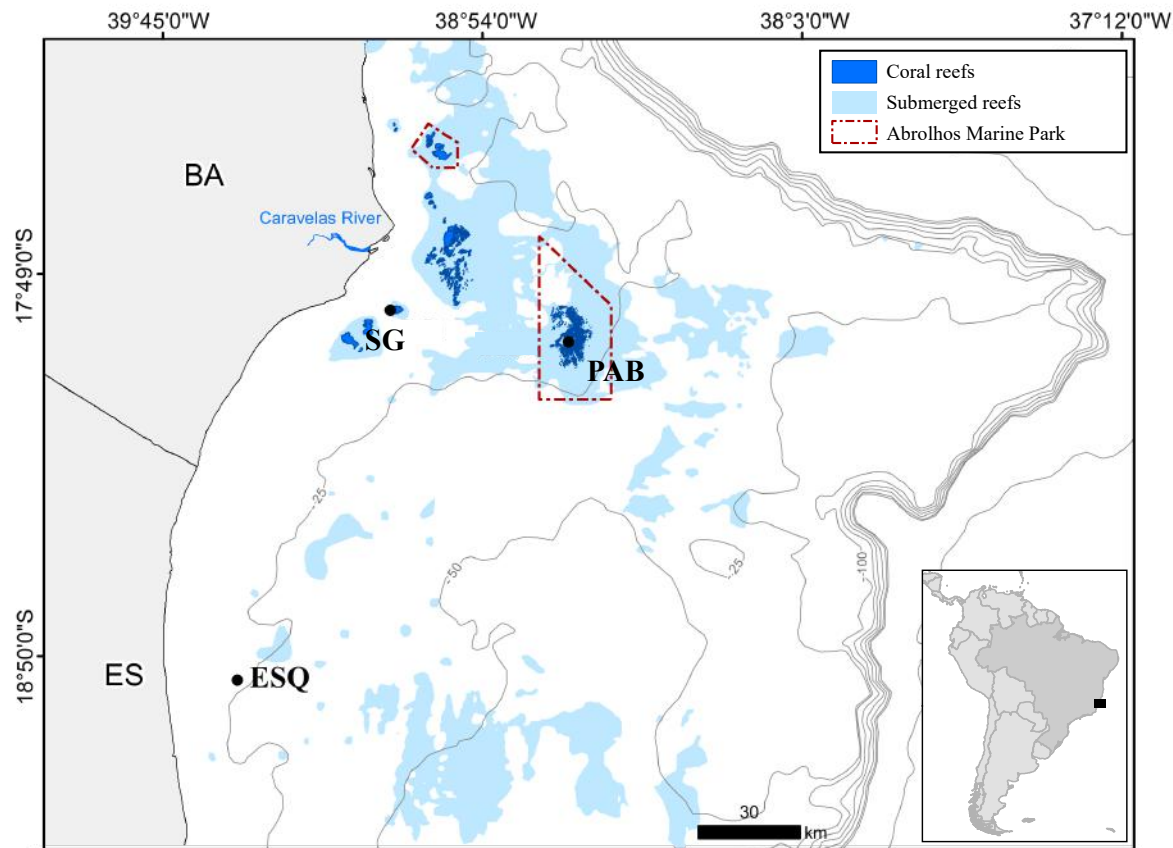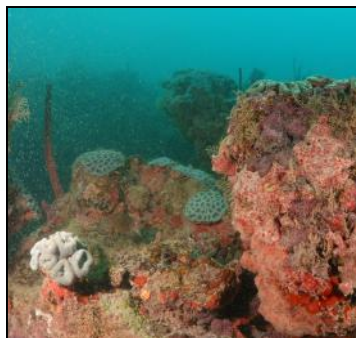

*Esquecidos Reef*

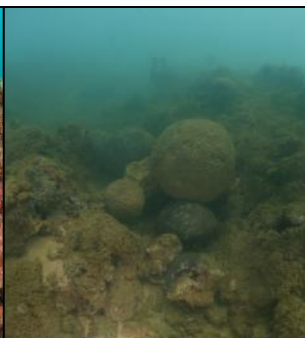

*Sebastião Gomes Reef*

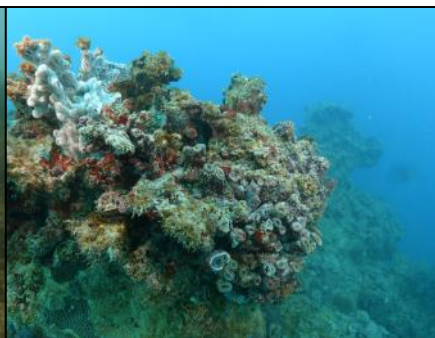

*Parcel dos Abrolhos Reef*

Supplement: Supplemental Information 3 — The top panel illustrates the locations of the Esquecidos Reef (ESQ), Sebastião Gomes Reef (SG), and Parcel dos Abrolhos Reef (PAB) within the Abrolhos Marine Park in Brazil. Coral reefs are marked in blue, submerged reefs are indicated, and the boundaries of the Abrolhos Marine Park are outlined in red. The inset shows the location of the Abrolhos Marine Park within Brazil. The bottom panel shows the benthic community and structure found in each reef. Photo credit: Rodrigo Leão de Moura. Source of Brazilian coastline: IBGE/DGC. Base Cartográfica Contínua do Brasil, 1:250.000 – BC250: 2017 version . Rio de Janeiro, 2017. [file peerj-13-20116-s003.pdf]
