# Supplementary material for: Hidden microalgae diversity in reef systems: reanalysis of coral microbiomes reveals spatial patterns of coral-associated plastid communities in the Southwestern Atlantic Ocean (SWAO)
Source: PeerJ. 2025 Nov 3;13:e20116. doi: 10.7717/peerj.20116 (PMC12591052; doi:10.7717/peerj.20116)
Supplement: Supplemental Information 7 [file peerj-13-20116-s007.docx]

**Statistical tests results used in Abrolhos reefs**

F = 54.268, num df = 1.000, denom df = 32.895, p-value = 1.884e-08

**Table S4:** Welch’s test of Shannon diversity of environmental matrix samples in Abrolhos reefs.

|  | **Df** | **Sum Sq** | **Mean Sq** | **F value** | **Pr(>F)** |  |
| --- | --- | --- | --- | --- | --- | --- |
| sampling_reef | 2 | 2.360 | 1.1801 | 5.226 | 0.01702 | * |
| Tempo3 | 2 | 0.363 | 0.1817 | 0.805 | 0.46361 |  |
| sampling_reef:time | 2 | 4.085 | 2.0423 | 9.044 | 0.00211 | ** |
| Residuals | 17 | 3.839 | 0.2258 |  |  |  |

**Table S5:** Anova (aov) of Shannon diversity of *M. harttii* samples in Abrolhos reefs.

$emmeans

Tempo4 = Apr22:

sampling_reef emmean SE df lower.CL upper.CL

ESQ nonEst NA NA NA NA

PAB 0.776 0.238 17 0.275 1.28

SG 1.818 0.238 17 1.316 2.32

Tempo4 = May21:

sampling_reef emmean SE df lower.CL upper.CL

ESQ 0.755 0.274 17 0.176 1.33

PAB 1.557 0.274 17 0.978 2.14

SG nonEst NA NA NA NA

Tempo4 = Sep21:

sampling_reef emmean SE df lower.CL upper.CL

ESQ 0.444 0.274 17 -0.135 1.02

PAB 1.859 0.274 17 1.281 2.44

SG 0.798 0.238 17 0.297 1.30

Confidence level used: 0.95

$contrasts

Tempo4 = Apr22:

contrast estimate SE df t.ratio p.value

ESQ - PAB nonEst NA NA NA NA

ESQ - SG nonEst NA NA NA NA

PAB - SG -1.042 0.336 17 -3.101 0.0065 **

Tempo4 = May21:

contrast estimate SE df t.ratio p.value

ESQ - PAB -0.802 0.388 17 -2.066 0.0544 .

ESQ - SG nonEst NA NA NA NA

PAB - SG nonEst NA NA NA NA

Tempo4 = Sep21:

contrast estimate SE df t.ratio p.value

ESQ - PAB -1.416 0.388 17 -3.649 0.0053 **

ESQ - SG -0.354 0.363 17 -0.976 0.6012

PAB - SG 1.061 0.363 17 2.925 0.0244 .

Signif. codes: 0 ‘***’ 0.001 ‘**’ 0.01 ‘*’ 0.05 ‘.’ 0.1 ‘ ’ 1

P value adjustment: tukey method for varying family sizes

**Table S6:** Pairwise comparisons of Abrolhos *M. harttii* samples’ Shannon diversity among reef sites within each sampling period.

|  | **Df** | **SumOfSqs** | **R2** | **F** | **Pr(>F)** |  |
| --- | --- | --- | --- | --- | --- | --- |
| matrix | 1 | 231.24 | 0.16972 | 9.6465 | 0.001 | *** |
| sampling_reef | 2 | 167.62 | 0.12303 | 3.4962 | 0.001 | *** |
| time | 2 | 73.83 | 0.05419 | 1.5401 | 0.027 | * |
| sampling_reef:time | 2 | 74.75 | 0.05487 | 1.5592 | 0.026 | * |
| Residual | 34 | 815.03 | 0.59820 |  |  |  |
| Total | 41 | 1362.47 | 1 |  |  |  |

**Table S7:** Permanova (adonis2) of environmental matrix communities in Abrolhos reefs.

| **Group** | **Species** | **Stat** | **P.value** | **Signif.** |
| --- | --- | --- | --- | --- |
| coral | Eukaryota.Archaeplastida.Chlorophyta.Ulvophyceae.Bryopsidales.Bryopsidales_X.Ostreobium | 0.957 | 0.001 | *** |
| coral | Eukaryota.Alveolata.Apicomplexa.Colpodellidea.Colpodellida.Colpodellaceae.Colpodellidae | 0.842 | 0.001 | *** |
| coral | Eukaryota.Archaeplastida.Rhodophyta.Florideophyceae.Corallinales.Corallinales_X.Calliarthron | 0.842 | 0.001 | *** |
| coral | Eukaryota.Stramenopiles.Ochrophyta.Bacillariophyta.Bacillariophyta_X.Raphid.pennate.Raphid.pennate_X | 0.842 | 0.001 | *** |
| coral | Eukaryota.Archaeplastida.Chlorophyta.Ulvophyceae.Bryopsidales.Bryopsidales_X.Bryopsidales_XX | 0.791 | 0.001 | *** |
| coral | Eukaryota.Stramenopiles.Ochrophyta.Bacillariophyta.Bacillariophyta_X.Raphid.pennate.Navicula | 0.744 | 0.004 | ** |
| coral | Eukaryota.Archaeplastida.Rhodophyta.Florideophyceae.Corallinales.Corallinales_X.__ | 0.645 | 0.002 | ** |
| coral | Eukaryota.Stramenopiles.Ochrophyta.Phaeophyceae.Phaeophyceae_X.Phaeophyceae_XX.Ectocarpus | 0.575 | 0.019 | * |
| coral | Eukaryota.Archaeplastida.Rhodophyta.Florideophyceae.__.__.__ | 0.54 | 0.04 | * |
| coral | Eukaryota.Archaeplastida.Chlorophyta.Ulvophyceae.Bryopsidales.Bryopsidales_X.Bryopsis | 0.5 | 0.042 | * |
| coral | Eukaryota.Stramenopiles.Ochrophyta.Bacillariophyta.Bacillariophyta_X.Raphid.pennate.Amphora | 0.5 | 0.041 | * |
| water | Eukaryota.Stramenopiles.Ochrophyta.Pelagophyceae.Pelagomonadales.Pelagomonadaceae.Aureococcus | 0.998 | 0.001 | *** |
| water | Eukaryota.Hacrobia.Cryptophyta.Cryptophyceae.Cryptomonadales.Cryptomonadales_X.Teleaulax | 0.996 | 0.001 | *** |
| water | Eukaryota.Stramenopiles.Ochrophyta.Dictyochophyceae.Dictyochophyceae_X.Dictyochophyceae_XX.Dictyochophyceae_XXX | 0.994 | 0.001 | *** |
| water | Eukaryota.Stramenopiles.Ochrophyta.Bacillariophyta.Bacillariophyta_X.Polar.centric.Mediophyceae.__ | 0.958 | 0.001 | *** |
| water | Eukaryota.Hacrobia.Haptophyta.Prymnesiophyceae.Phaeocystales.Phaeocystaceae.Phaeocystis | 0.931 | 0.001 | *** |
| water | Eukaryota.Stramenopiles.Ochrophyta.Chrysophyceae.Chrysophyceae_X.Chrysophyceae_XX.Chrysophyceae_XXX | 0.913 | 0.001 | *** |
| water | Eukaryota.Stramenopiles.Ochrophyta.Bacillariophyta.Bacillariophyta_X.Bacillariophyta_XX.Bacillariophyta_XXX | 0.892 | 0.001 | *** |
| water | Eukaryota.Stramenopiles.Ochrophyta.Bacillariophyta.Bacillariophyta_X.Polar.centric.Mediophyceae.Thalassiosira | 0.888 | 0.001 | *** |
| water | Eukaryota.Archaeplastida.Chlorophyta.Pyramimonadales.Pyramimonadales_X.Pyramimonadales_XX.Pyramimonas | 0.85 | 0.001 | *** |
| water | Eukaryota.Stramenopiles.Ochrophyta.Bacillariophyta.Bacillariophyta_X.Polar.centric.Mediophyceae.Chaetoceros | 0.848 | 0.001 | *** |
| water | Eukaryota.Stramenopiles.Ochrophyta.Xanthophyceae.Xanthophyceae_X.Xanthophyceae_XX.Vaucheria | 0.846 | 0.001 | *** |
| water | Eukaryota.Stramenopiles.Ochrophyta.Bacillariophyta.Bacillariophyta_X.Raphid.pennate.Cylindrotheca | 0.825 | 0.001 | *** |
| water | Eukaryota.Hacrobia.Haptophyta.Prymnesiophyceae.Prymnesiophyceae_X.Prymnesiaceae | 0.816 | 0.001 | *** |
| water | Eukaryota.Archaeplastida.Chlorophyta.Chloropicophyceae.Chloropicales.Chloropicaceae.Chloropicon | 0.786 | 0.002 | ** |
| water | Eukaryota.Archaeplastida.Chlorophyta.Mamiellophyceae.Mamiellales.Bathycoccaceae.Ostreococcus | 0.782 | 0.001 | *** |
| water | Eukaryota.Stramenopiles.Ochrophyta.Bacillariophyta.Bacillariophyta_X.Polar.centric.Mediophyceae.Polar.centric.Mediophyceae_X | 0.772 | 0.001 | *** |
| water | Eukaryota.Stramenopiles.Ochrophyta.Pinguiophyceae.Pinguiochrysidales.Pinguiochrysidaceae.Phaeomonas | 0.705 | 0.001 | *** |
| water | Eukaryota.Archaeplastida.Chlorophyta.Nephroselmidophyceae.Nephroselmidales.Nephroselmidales_X.Nephroselmis | 0.667 | 0.001 | *** |
| water | Eukaryota.Archaeplastida.Chlorophyta.Palmophyllophyceae.Prasinococcales.Prasinococcales.Clade.B.Prasinoderma | 0.667 | 0.001 | *** |
| water | Eukaryota.Stramenopiles.Ochrophyta.Eustigmatophyceae.Eustigmatophyceae_X.Eustigmatophyceae_XX.Nannochloropsis | 0.642 | 0.005 | ** |
| water | Eukaryota.Archaeplastida.Chlorophyta.Chlorodendrophyceae.Chlorodendrales.Chlorodendraceae.Tetraselmis | 0.619 | 0.002 | ** |
| water | Eukaryota.Excavata.Discoba.Euglenozoa.Euglenida.Eutreptiales.Eutreptiella | 0.577 | 0.003 | ** |
| water | Eukaryota.Hacrobia.Cryptophyta.Cryptophyceae.Cryptomonadales.Cryptomonadales_X.Proteomonas | 0.577 | 0.004 | ** |
| water | Eukaryota.Hacrobia.Haptophyta.Prymnesiophyceae.Prymnesiophyceae_X.__.__ | 0.577 | 0.007 | ** |
| water | Eukaryota.Stramenopiles.Ochrophyta.Bacillariophyta.Bacillariophyta_X.Radial.centric.basal.Coscinodiscophyceae.Rhizosolenia | 0.577 | 0.002 | ** |
| water | Eukaryota.Stramenopiles.Ochrophyta.Dictyochophyceae.Dictyochophyceae_X.Florenciellales.Florenciella | 0.527 | 0.013 | * |
| water | Eukaryota.Archaeplastida.Chlorophyta.Mamiellophyceae.Mamiellales.Mamiellaceae.Mantoniella | 0.515 | 0.03 | * |
| water | Eukaryota.Hacrobia.Haptophyta.Pavlovophyceae.Pavlovales.Pavlovaceae.Pavlova | 0.505 | 0.036 | * |
| water | Eukaryota.Stramenopiles.Ochrophyta.Dictyochophyceae.Dictyochophyceae_X.Pedinellales.Mesopedinella | 0.471 | 0.031 | * |

**Table S8:** Indicator species analysis (indcspecies) of the environmental matrix in Abrolhos reefs.

|  | **Df** | **SumOfSqs** | **R2** | **F** | **Pr(>F)** |  |
| --- | --- | --- | --- | --- | --- | --- |
| sampling_reef | 2 | 209.44 | 0.31890 | 5.0330 | 0.001 | *** |
| time | 2 | 36.14 | 0.05503 | 0.8685 | 0.625 |  |
| sampling_reef:time | 2 | 57.47 | 0.08750 | 1.3810 | 0.114 |  |
| Residual | 17 | 353.72 | 0.53857 |  |  |  |
| Total | 23 | 656.78 | 1 |  |  |  |

**Table S9:** Permanova (adonis2) of coral communities in Abrolhos reefs.

| **Group** | **Species** | **Stat** | **P.value** | **Significance** |
| --- | --- | --- | --- | --- |
| ESQ | Eukaryota.Archaeplastida.Rhodophyta.Florideophyceae.Corallinales.Corallinales_X.__ | 0.971 | 0.001 | *** |
| ESQ | Eukaryota.Archaeplastida.Chlorophyta.Chloropicophyceae.Chloropicales.Chloropicaceae.Chloropicon | 0.691 | 0.019 | * |
| PAB+SG | Eukaryota.Archaeplastida.Chlorophyta.Ulvophyceae.Bryopsidales.Bryopsidales_X.Ostreobium | 0.987 | 0.002 | ** |
| PAB+SG | Eukaryota.Alveolata.Apicomplexa.Colpodellidea.Colpodellida.Colpodellaceae.Colpodellidae | 0.909 | 0.015 | * |
| PAB+SG | Eukaryota.Archaeplastida.Chlorophyta.Ulvophyceae.Bryopsidales.Bryopsidales_X.Bryopsidales_XX | 0.881 | 0.005 | ** |

**Table S10:** Indicator species analysis (indcspecies) of *M. harttii* samples in Abrolhos reefs.

|  | **Df** | **Sum Sq** | **Mean Sq** | **F value** | **Pr(>F)** |  |
| --- | --- | --- | --- | --- | --- | --- |
| sampling_reef | 2 | 0.3109 | 0.1555 | 22.63 | 0.000126 | *** |
| Tempo3 | 2 | 0.3189 | 0.1595 | 23.21 | 0.000113 | *** |
| sampling_reef:Tempo3 | 2 | 0.7266 | 0.3633 | 52.89 | 2.28e-06 | *** |
| Residuals | 11 | 0.0756 | 0.0069 |  |  |  |

**Table S11:** Anova (aov) of Shannon diversity of seawater samples in Abrolhos reefs.

$emmeans

Tempo4 = Apr22:

sampling_reef emmean SE df lower.CL upper.CL

ESQ nonEst NA NA NA NA

PAB 2.29 0.0479 11 2.19 2.40

SG 2.32 0.0586 11 2.19 2.44

Tempo4 = May21:

sampling_reef emmean SE df lower.CL upper.CL

ESQ 2.61 0.0479 11 2.51 2.72

PAB 1.84 0.0479 11 1.74 1.95

SG nonEst NA NA NA NA

Tempo4 = Sep21:

sampling_reef emmean SE df lower.CL upper.CL

ESQ 2.24 0.0829 11 2.06 2.42

PAB 2.60 0.0479 11 2.49 2.70

SG 2.06 0.0479 11 1.96 2.17

Confidence level used: 0.95

$contrasts

Tempo4 = Apr22:

contrast estimate SE df t.ratio p.value

ESQ - PAB nonEst NA NA NA NA

ESQ - SG nonEst NA NA NA NA

PAB - SG -0.0225 0.0757 11 -0.298 0.7715

Tempo4 = May21:

contrast estimate SE df t.ratio p.value

ESQ - PAB 0.7724 0.0677 11 11.414 <.0001 ***

ESQ - SG nonEst NA NA NA NA

PAB - SG nonEst NA NA NA NA

Tempo4 = Sep21:

contrast estimate SE df t.ratio p.value

ESQ - PAB -0.3527 0.0957 11 -3.685 0.0092 **

ESQ - SG 0.1802 0.0957 11 1.883 0.1894

PAB - SG 0.5329 0.0677 11 7.875 <.0001 ***

Signif. codes: 0 ‘***’ 0.001 ‘**’ 0.01 ‘*’ 0.05 ‘.’ 0.1 ‘ ’ 1

P value adjustment: tukey method for varying family sizes.

**Table S12:** Pairwise comparisons of Abrolhos water samples’ Shannon diversity among reef sites within each sampling period.

|  | **Df** | **SumOfSqs** | **R2** | **F** | **Pr(>F)** |  |
| --- | --- | --- | --- | --- | --- | --- |
| sampling_reef | 2 | 112.44 | 0.23699 | 3.6743 | 0.001 | *** |
| time | 2 | 109.47 | 0.23072 | 3.5771 | 0.001 | *** |
| sampling_reef:time | 2 | 84.24 | 0.17755 | 2.7527 | 0.001 | *** |
| Residual | 11 | 168.31 | 0.35474 |  |  |  |
| Total | 17 | 474.46 | 1 |  |  |  |

**Table S13:** Permanova (adonis2) of seawater communities in Abrolhos reefs.

| **Group** | **Species** | **Stat** | **P.value** | **Significance** |
| --- | --- | --- | --- | --- |
| ESQ | Eukaryota.Archaeplastida.Chlorophyta.Chloropicophyceae.Chloropicales.Chloropicaceae.Chloroparvula | 0.866 | 0.007 | ** |
| ESQ | Eukaryota.Stramenopiles.Ochrophyta.Pelagophyceae.Sarcinochrysidales.Sarcinochrysidaceae.Sarcinochrysidaceae_X | 0.866 | 0.007 | ** |
| ESQ | Eukaryota.__.__.__.__.__.__ | 0.707 | 0.038 | * |
| ESQ | Eukaryota.Hacrobia.__.__.__.__.__ | 0.707 | 0.043 | * |
| ESQ | Eukaryota.Stramenopiles.Ochrophyta.Chrysophyceae.Chrysophyceae_X.__.__ | 0.707 | 0.039 | * |
| PAB | Eukaryota.Stramenopiles.Ochrophyta.Pinguiophyceae.Pinguiochrysidales.Pinguiochrysidaceae.Phaeomonas | 0.888 | 0.007 | ** |
| PAB | Eukaryota.Hacrobia.Cryptophyta.Cryptophyceae.Cryptomonadales.Cryptomonadales_X.Proteomonas | 0.816 | 0.016 | * |
| PAB | Eukaryota.Stramenopiles.Ochrophyta.Bacillariophyta.Bacillariophyta_X.Radial.centric.basal.Coscinodiscophyceae.Rhizosolenia | 0.816 | 0.022 | * |
| PAB | Eukaryota.Archaeplastida.Chlorophyta.Chlorodendrophyceae.Chlorodendrales.Chlorodendraceae.Tetraselmis | 0.796 | 0.019 | * |
| ESQ+SG | Eukaryota.Stramenopiles.Ochrophyta.Bacillariophyta.Bacillariophyta_X.Raphid.pennate.Cylindrotheca | 0.99 | 0.001 | *** |
| ESQ+SG | Eukaryota.Stramenopiles.Ochrophyta.Xanthophyceae.Xanthophyceae_X.Xanthophyceae_XX.Vaucheria | 0.985 | 0.001 | *** |
| ESQ+SG | Eukaryota.Archaeplastida.Chlorophyta.Chloropicophyceae.Chloropicales.Chloropicaceae.Chloropicon | 0.9 | 0.041 | * |
| ESQ+SG | Eukaryota.Stramenopiles.Ochrophyta.Bacillariophyta.Bacillariophyta_X.Raphid.pennate.__ | 0.858 | 0.014 | * |
| ESQ+SG | Eukaryota.Hacrobia.Haptophyta.Prymnesiophyceae.Prymnesiophyceae_X.__.__ | 0.739 | 0.046 | * |

**Table S14:** Indicator species analysis (indcspecies) of seawater samples in Abrolhos reefs.
