# Supplementary material for: Hidden microalgae diversity in reef systems: reanalysis of coral microbiomes reveals spatial patterns of coral-associated plastid communities in the Southwestern Atlantic Ocean (SWAO)
Source: PeerJ. 2025 Nov 3;13:e20116. doi: 10.7717/peerj.20116 (PMC12591052; doi:10.7717/peerj.20116)
Supplement: Supplemental Information 9 [file peerj-13-20116-s009.pdf]

Systematic Review Rationale

The rationale for conducting this systematic review and re-analysis is rooted in the need to address significant gaps in the current understanding of the role of plastid-bearing eukaryotes in coral microbiomes of the Southwestern Atlantic Ocean (SAO). While numerous studies have examined bacterial components of coral microbiomes as well as Symbiodiniaceae, there is a lack of comprehensive analysis focusing on the microalgae communities, despite the increase in attention to these microorganisms in recent years. Our re-analysis based on the plastidial 16S sequence database offers a straightforward bioinformatic pipeline to analyze microalgae communities in coral tissues. Given the overwhelming abundance of coral and Symbiodiniaceae genetic material in environmental coral samples, this data would be hardly obtained using eukaryotic markers (e.g. 18S amplicons).

This re-analysis contributes to knowledge by providing a detailed inventory of plastid-bearing eukaryotes occurrences from plastid 16S rRNA sequences from over 200 samples, distinguishing between plastid communities in coral tissue and surrounding seawater. By compiling data from 20 studies, this work offers a comparative analysis that allows for an evaluation of the observed microalgae based on its prevalence in coral samples, revealing not only the known and widespread, but also less prevalent coral-associated microalgae. Moreover, although plastid sequences have already been used to evaluate the occurrence of widespread plastid-bearing eukaryotes, such as corallicolids and *Ostreobium*, an evaluation of the factors influencing coral-associated plastids at the ecological community level has not been done yet. Our approach reveals that plastid communities can serve as biomarkers of temporal and geographical variations in coral microbiomes.

In this sense, this study builds on previous research by incorporating overlooked plastid sequences through bioinformatic methods. These findings have the potential to deepen our understanding of holobiont dynamics and the interactions between corals and their microbiomes, thereby enhancing insights into coral resilience against environmental stressors.
